# Generation and characterization of chimeric Tembusu viruses containing pre-membrane and envelope genes of Japanese encephalitis virus

**DOI:** 10.3389/fmicb.2023.1140141

**Published:** 2023-06-22

**Authors:** Bangfeng Xu, Xingpo Liu, Dawei Yan, Qiaoyang Teng, Chunxiu Yuan, Zhifei Zhang, Qinfang Liu, Zejun Li

**Affiliations:** Department of Avian Infectious Diseases, Shanghai Veterinary Research Institute, Chinese Academy of Agricultural Sciences, Shanghai, China

**Keywords:** TMUV, JEV, Flavivirus, attenuated vaccines, virulence

## Abstract

Since its outbreak in 2010, Tembusu virus (TMUV) has spread widely throughout China and Southeast Asia, causing significant economic losses to the poultry industry. In 2018, an attenuated vaccine called FX2010-180P (180P) was licensed for use in China. The 180P vaccine has demonstrated its immunogenicity and safety in mice and ducks. The potential use of 180P as a backbone for flavivirus vaccine development was explored by replacing the pre-membrane (prM) and envelope (E) genes of the 180P vaccine strain with those of Japanese encephalitis virus (JEV). Two chimeric viruses, 180P/JEV-prM-E and 180P/JEV-prM-E_S156P_ with an additional E protein S156P mutation were successfully rescued and characterized. Growth kinetics studies showed that the two chimeric viruses replicated to similar titers as the parental 180P virus in cells. Animal studies also revealed that the virulence and neuroinvasiveness of the 180P/JEV-prM-E chimeric virus was decreased in mice inoculated intracerebrally (i.c.) and intranasally (i.n.), respectively, compared to the wild-type JEV strain. However, the chimeric 180P/JEV-prM-E virus was still more virulent than the parent 180P vaccine in mice. Additionally, the introduction of a single E_S156P_ mutation in the chimeric virus 180P/JEV-prM-E_S156P_ further attenuated the virus, which provided complete protection against challenge with a virulent JEV strain in the mouse model. These results indicated that the FX2010-180P could be used as a promising backbone for flavivirus vaccine development.

## 1. Introduction

The Flaviviridae family consists mainly of zoonotic pathogens that infect both humans and animals, such as Japanese encephalitis virus (JEV), dengue virus (DENV), Zika virus (ZIKV), and West Nile virus (WNV) (Mackenzie et al., 2004; Monath and Vasconcelos, 2015; Pierson and Diamond, 2020). The *Flavivirus* is single-stranded positive RNA virus with a genome of about ~11 kb, composed of a 5′-UTR, an ORF region encodes polyprotein, and a 3′-UTR. The encoded polyprotein is cleaved into three structural proteins (capsid C, pre-membrane prM, and envelope E) and seven non-structural proteins by viral and host proteases (Chambers et al., 1990). The structural proteins of *Flavivirus* are involved in attachment, entry, and virion formation (Allison et al., 2001; Lorenz et al., 2002), while the nonstructural proteins are responsible for genome replication, virion assembly, and the evasion of host antiviral responses (Umareddy et al., 2006; Miller et al., 2007; Leung et al., 2008). Previous research have shown that the prM/E proteins are critical for neurovirulence of *Flavivirus* (Leng et al., 2020), and the E protein is involved in binding to receptors of the host cell surface. Glycosylation of the Envelope protein has been identified as a virulence determinant for several flaviviruses (Carbaugh and Lazear, 2020). The E protein is also a prime target for vaccine development against *Flavivirus.*

TMUV, a member of the *Flavivirus* genus in the Flaviviridae family, was first isolated from mosquitoes in Malaysia in 1955 (Platt et al., 1975). In 2010, an outbreak of TMUV occurred in China, resulting in major clinical symptoms such as growth retardation, severe reduction in egg production, and neurological symptoms (Yan et al., 2011). Since then, the virus has rapidly spread among duck farms in China and Southeast Asia, becoming endemic (Cao et al., 2011; Su et al., 2011; Tang et al., 2013; Thontiravong et al., 2015). To control the spread and prevalence of TMUV in duck population, an attenuated vaccine FX2010-180P (180P) was developed through serial passaging of a wildtype TMUV-FX2010 on chicken embryo fibroblasts (CEF) (Li et al., 2014). Subsequent studies showed that the 180P was attenuated in mice and ducks (Li et al., 2014), with no clinical symptoms or tissue damage observed in ducks infected by a high-dose inoculation (Li et al., 2014). Moreover, a low-dose 180P elicited good immunogenicity in ducks and provided complete protection against challenge with a virulent strain (Li et al., 2014), which indicated that the 180P was an ideal attenuated vaccine strain. As a result, the 180P vaccine was licensed in China in 2018 and is now widely used in ducks to prevent the TMUV infection.

To investigate whether the 180P could serve as a backbone for vaccine development against other *Flavivirus*, the prM and E genes of 180P were replaced by those of a wildtype JEV virus. A chimeric virus 180P/JEV-prM-E was successfully rescued in the background of the 180P. It was found that the 180P/JEV-prM-E was attenuated compared with the wildtype JEV, with a mortality rate of 60% of mice inoculated intracerebrally. To further attenuate the 180P/JEV-prM-E, a substitution of S156P was introduced into the E protein of the 180P/JEV-prM-E, 180P/JEV-prM-E_S156P_ were significantly attenuated in mice compared with the 180P/JEV-prM-E, and provided complete protection against challenge with a wildtype JEV in mouse model, which suggested that the 156S of prM-E is critical for the virulence of JEV. Overall, this study presents a novel approach for developing vaccines against Flaviviruses.

## 2. Materials and methods

### 2.1. Cells and viruses

Baby Hamster Syrian Kidney cells (BHK-21) and Vero-E6 cells were obtained from the American Type Culture Collection (ATCC) and maintained in Dulbecco’s Modified Eagle Medium (DMEM) (Hyclone, Logan, UT, United States), supplemented with 10% fetal bovine serum (FBS) (Biowest, South America origin, Riverside, MO, United States), 100 U/mL of penicillin, and 100 μg/mL of streptomycin (Invitrogen, Carlsbad, CA, United States) at 37°C in a 5% CO_2_ humidified incubator. The FX2010-180P vaccine strain, wildtype JEV virus (HEN0701, with the GenBank access FJ495189.1, which belonged to the JEV genotype I group) and JEV attenuated vaccine SA14-14-2 was prepared as described previously (Li et al., 2014). The prM and E genes of 180P/JEV-prM-E virus were derived from JEV isolates ZJ14-52 (GenBank access KM576778.1). All of the rescued viruses were propagated once on BHK-21 cells, aliquoted, and stored at −80°C.

### 2.2. Plasmid construction

To generate the templates for PCR-based virus rescue, four plasmids p180PT7-1-976, p180PE957–2459, p180P2433–3831, and p180P3656-10991 containing the overlapping fragments of the FX2010-180P were generated and were used as templates for full-length genome amplification (Yan et al., 2022). To rescue the 180P/JEV-prM-E and 180P/JEV-prM-E_S156P_ viruses, the plasmids p180PT7–1-455, pJEV-prM-E, and pJEV-prM-E_S156P_ were constructed, respectively.

### 2.3. PCR-based rescue of chimeric viruses

The reverse genetics system of the 180P was prepared as described previously (Yan et al., 2018). Briefly, p180PT7-1-976, p180PE957-2459, p180P2433-3831, and p180P3656-10991 plasmids were used as templates to amplify four overlapping fragments, and the full-length cDNA of 180P with T7 promoter was produced by fusion-PCR using the four overlapping fragments as described previously (Yan et al., 2022). To generate the infectious viral RNAs, the cDNAs were transcribed *in vitro* using mMESSAGEmMACHINE^®^ T7 Kit (Invitrogen, Carlsbad, CA, United States) and purified by lithium chloride precipitation. The RNAs were then transfected into BHK-21 cells at 70%–80% confluency, cultured in T25 cm^2^ flasks, with an amount of 5 μg using Lipofectamine LTX& Plus Reagent (Invitrogen, Carlsbad, CA, United States). The cell culture medium was changed to DMEM containing 2% FBS at 6 h post-transfection. The virus released into the supernatant was collected when 70%–80% transfected cells start showing apparent cytopathic effects (CPEs). To rescue the 180P/JEV-prM-E or single-site mutant chimeric 180P/JEV-prM-E_S156P_ viruses, plasmids p180PT7–1-976 and p180PE957-2459 were replaced by the p180PT7-1-455, pJEV-prM-E, and pJEV-prM-E_S156P_ plasmids during rescues, respectively. The rescued viruses were amplified on BHK-21 cells for further study (Zhang et al., 2020). The sequences of the recused viruses were confirmed by sanger sequencing.

### 2.4. Growth kinetics of chimeric viruses on BHK-21 and Vero cells

To determine the viral replication of rescued viruses *in vitro*, BHK-21 and Vero-E6 cells were prepared and cultured in T25 flasks. The cells were infected with the chimeric viruses and parental viruses at a multiplicity of infection (MOI) of 0.001. The infected cells were incubated with DMEM containing 2% FBS at 37°C, 5% CO_2_. The supernatant was collected every 12 h post-infection and titrated on BHK-21 cells.

### 2.5. Morphological observation of 180P/JEV-prM-E_S156P_ virus plaque

The 180P/JEV-prM-E_S156P_ mutant virus and its parental virus (180P/JEV-prM-E) were used to infect BHK-21 cells at 0.0001 MOI. The cells were incubated for 2 h at 37°C and 5% CO_2_. An equal volume of agarose and 2× DMEM were added to each well, fully mixed, and allowed to solidify at 4°C. The plates were then inverted and incubated at 37°C and 5% CO_2_ for 4 days. After formaldehyde fixation and crystal violet staining, typical plaque formation was observed at the appropriate amount of virus infection. Additionally, the diameter of 10 randomly selected plaques was measured for statistical analysis.

### 2.6. Animal experiments

To test the virulence and neuroinvasiveness of parental 180P, wildtype JEV, JEV attenuated vaccine SA14-14-2, and the 180P/JEV-prM-E and 180P/JEV-prM-E_S156P_ chimeric viruses in mouse, groups of 3 weeks-old female BALB/c mice were inoculated intracerebrally (i.c.), intranasally (i.n.), or intraperitoneally (i.p.) with 10^4.0^ TCID_50_ of each virus at volume of 30 μL(or at volume of 50 μL for 180P/JEV-prM-E_S156P_), 30 μL (or at volume of 50 μL for 180P/JEV-prM-E_S156P_), and 100 μL, respectively. And the control group was inoculated with PBS. In each group, three mice were euthanized on 4, 6, and 8 dpi by CO_2_ inhalation, respectively, and the tissue samples of brain, liver, spleen, lung, and kidney were collected for viral titration. Body weight data, survival data and clinical scores (weight loss and depression = 1, fluffy fur = 2, loss of appetite = 3 and death = 4) of the remaining five mice in each group were recorded daily until 14 dpi. And the neurovirulence and neuroinvasiveness of each virus were evaluated based on all the data.

To test the efficacy of the 180P/JEV-prM-E_S156P_ in mice, two groups of eight 3-week-old female BALB/c mice were intranasally immunized with 10^4.0^ TCID_50_ of 180P/JEV-prM-E_S156P_ mutant virus at volume of 50 μL and JEV attenuated vaccine SA14-14-2 at volume of 30 μL. Another control groups of consisting of eight mice were used as a negative control group (non-vaccinated mice; challenged). On day 21 post-inoculation, mouse blood was collected and serum was separated. To detect the JEV and TMUV specific antibodies in serum, IFA was conducted on the BHK-21 cells transfected with JEV E, TMUV C, prM and E protein expression plasmids. All animals were then challenged intranasally with 10^4.0^ TCID_50_ of wildtype JEV. Three mice were euthanized on 6 dpi by CO_2_ inhalation and the tissue samples of brain, spleen and lung were collected for viral titration. Body weight data of the remaining five mice were recorded daily until 14 dpi.

All animal studies were approved by the institutional Animal Care and Use Committee of Shanghai Veterinary Research Institute (SHVRI) and conducted in the Biological Safety Level 2 (BSL2) facility with permit number was SHVRI-SZ-20200506-02.

### 2.7. Virus loads in mouse tissue samples

To determine the virus load, the mouse tissue samples were weighed and homogenized in sterile PBS to yield homogenates. Tissue homogenates were clarified by centrifugation at 12,000 × g, 4°C for 10 min, and 10-fold serially diluted supernatants were inoculated onto 70%–80% confluent BHK-21 cells in 96-well plates at 37°C for 2 h. After adsorption, cells were washed and cultured with DMEM containing 2% FBS at 37°C. The virus titer was calculated by the method of Reed-Muench.

### 2.8. Statistical analysis

Statistical analyses were performed using GraphPad Prism version 7.0 for Windows (GraphPad Software, San Diego, CA). Significant differences were calculated using Student’s *t*-test, posed ANOVA. *p* < 0.05 was considered to be significant.

## 3. Results

### 3.1. Construction and growth kinetics of chimeric viruses

**Figure 1:**
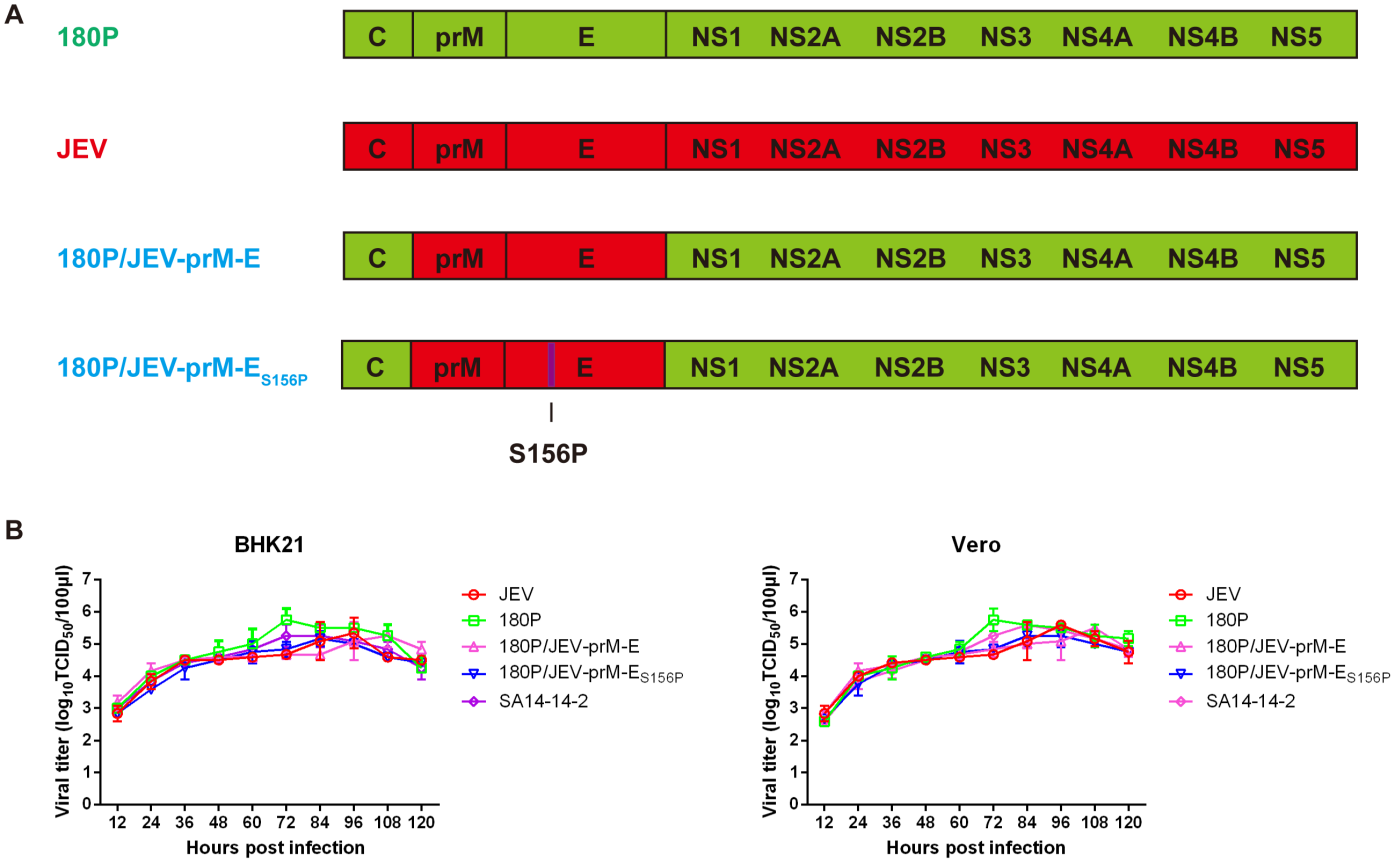
Virus generation, replication of parental, prM-E and prM-E_S156P_ switched chimeric 180P *in vitro*. **(A)** Schematic diagram of chimeric viruses 180P/JEV-prM-E and 180P/JEV-prM-E_S156P_. The colored bars indicate the origin of the viral protein: red, JEV; green, 180P. The viruses were rescued in the background of 180P. **(B)** Replication of parental and chimeric viruses on BHK-21 and Vero cells. The data for virus titers indicate the means of triplicates.

To investigate the potential of using 180P as a backbone for developing flavivirus vaccines, the 180P/JEV-prM-E chimeric virus containing prM and E genes of JEV were rescued in backbone of the 180P (Figure 1A). Growth kinetics showed that the 180P/JEV-prM-E had similar replication abilities to wildtype JEV and 180P on BHK-21 and Vero-E6 cells infected with each virus at an MOI of 0.001 (Figure 1B). The 180P/JEV-prM-E replicated to slightly lower titers than the 180P virus at 72 h post infection, which suggested that the JEV prM-E is compatible with the backbone of 180P, and did not affect the replication abilities of chimeric viruses significantly.

### 3.2. The 180P/JEV-prM-E was attenuated compared with the wildtype JEV in mice

**Figure 2:**
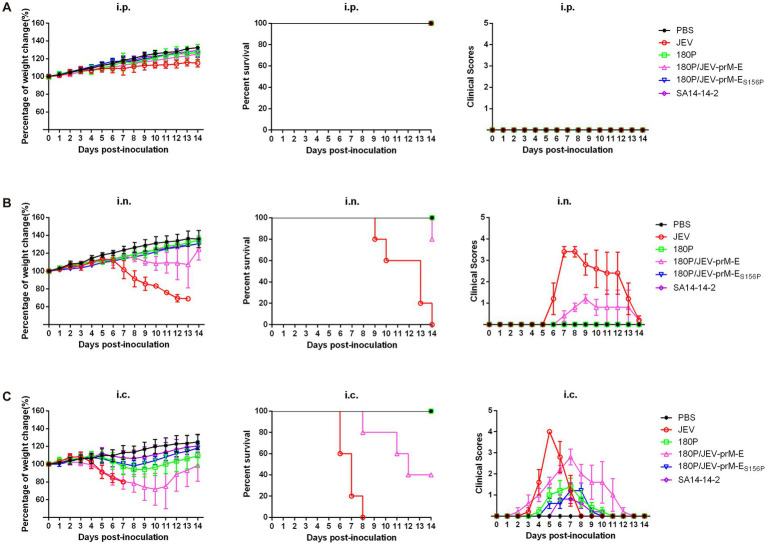
Virulence and neuroinvasiveness of chimeric viruses in mice. **(A)** Groups of five mice were i.p. inoculated with doses of 10^4^ TCID_50_ of viruses in a 100 μL volume. **(B)** Groups of five mice were i.n. inoculated with doses of 10^4^ TCID_50_ of viruses in a 30 μL volume (or at volume of 50 μL for 180P/JEV-prM-E_S156P_). **(C)** Groups of five mice were i.c. inoculated with doses of 10^4^ TCID_50_ of viruses in a 30 μL volume (or at volume of 50 μL for 180P/JEV-prM-E_S156P_). And the control group was inoculated with the same amount of sterile PBS. Body weight, survival data and clinical scores (weight loss and depression = 1, fluffy fur = 2, loss of appetite = 3, death = 4) of mice in each group were recorded daily until to 14 dpi.

To further evaluate the pathogenesis of 180P/JEV-prM-E in mice, neuroinvasive ability of the viruses were evaluated in mice inoculated intraperitoneally and intranasally. The results showed that intraperitoneal inoculation did not result in any weight loss or mortality in mice (Figure 2A), whereas intranasal inoculation with JEV caused severe weight loss and all mice died within 14 dpi (Figure 2B). In contrast, in the 180P/JEV-prM-E infected group, only one mouse died on 14 dpi, and slight body weight lost was observed (Figure 2B). The 180P infected mice did not show any clinical signs (Figure 2B). The clinical scores of neuroinvasiveness in the 180P/JEV-prM-E group was lower than that in the wildtype JEV group (Figure 2B). All the results indicated that the neuroinvasiveness of the 180P/JEV-prM-E was reduced significantly compared with the wildtype JEV.

Neurovirulence was evaluated through intracerebral inoculation, which resulted in weight loss in the 180P/JEV-prM-E group with 60% mortality on 14 dpi (Figure 2C). In contrast, the JEV caused 100% mortality within 8 dpi (Figure 2C), While the 180P infection only caused slight weight loss in mice without death (Figure 2C). The clinical scores of the wildtype JEV group were higher than that of the 180P/JEV-prM-E group. These results indicated that the neurovirulence of the 180P/JEV-prM-E virus was lower than that of the wildtype JEV.

**Figure 3:**
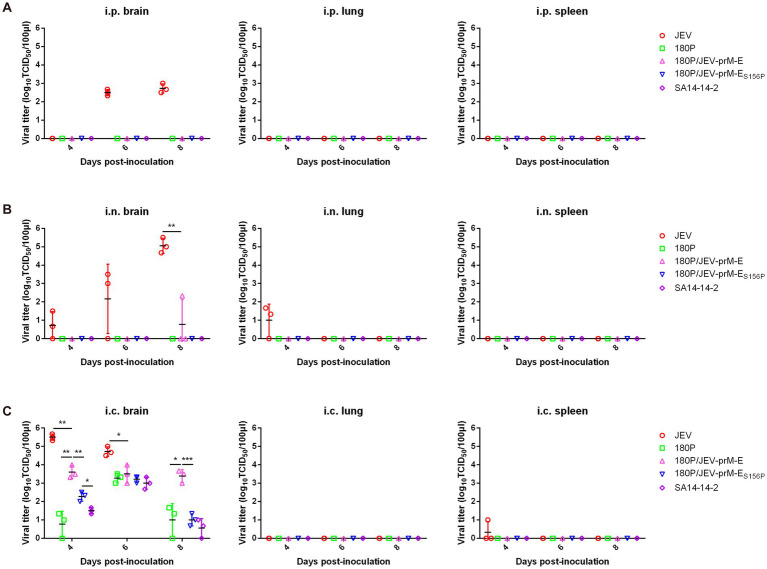
Distribution of virus in tissues under different inoculation methods at a dose of 10^4^ TCID_50_ of 180P, JEV and 180P/JEV-prM-E viruses. **(A)** Viral titers in brain, spleen and lung of i.p. inoculated mice were determined on BHK-21 cells at 4, 6, and 8 dpi. **(B)** Viral titers in brain, spleen and lung of i.n. inoculated mice were determined on BHK-21 cells at 4, 6, and 8 dpi. **(C)** Viral titers in brain, spleen and lung of i.c. inoculated mice were determined on BHK-21 cells at 4, 6, and 8 dpi. ^*^p < 0.05, ^**^p < 0.01, ^***^p < 0.001.

Virus distribution analysis in infected mice revealed that the viruses were mainly detected in the brains. In the i.p. group, only JEV was detected in brain of infected mice on 6 and 8 dpi, while no virus was detected in the spleen or lung tissues. Neither 180P/JEV-prM-E nor 180P were detected in the i.p. infected mice (Figure 3A). In the i.n. group, JEV was detected in the brains on 4, 6, 8 dpi and in the lung on 4 dpi. However, the 180P/JEV-prM-E was only detected in brain of one i.n. infected mouse. The 180P was not detected in any infected mice (Figure 3B). Furthermore, in i.c. group, virus was detected in the brains of the three virus infected groups, with the virus titers in the 180P/JEV-prM-E group lower than those in the JEV group, but higher than those in the 180P inoculated group (Figure 3C). Additionally, the virus was also detected in spleen of mice in the JEV group on 4 dpi (Figure 3C). Overall, these results indicated that the 180P/JEV-prM-E was attenuated compared with the wildtype JEV, but more virulent than the 180P in mice, and the prM-E protein is critical for the neurovirulence of JEV.

### 3.3. The JEV E S156P mutation further attenuates the chimeric virus 180P/JEV-prM-E in mice

Previous studies have identified the envelope protein glycosylation as a virulence determinant for multiple flaviviruses (Yan et al., 2018; Carbaugh and Lazear, 2020). In order to further attenuate the chimeric virus 180P/JEV-prM-E, the potential glycosylation site (154–156 NYS) on the JEV E protein was mutated to 154–156 NYP, and the chimeric virus 180P/JEV-prM-E_S156P_ with the glycosylation site deletion was successfully rescued (Figure 1A). The replication abilities of 180P/JEV-prM-E_S156P_ on BHK-21 and Vero-E6 cells were found to be similar to the parental chimeric virus 180P/JEV-prM-E (Figure 1B), which indicated that the glycosylation site deletion did not affect the viral replication abilities *in vitro*.

**Figure 4:**
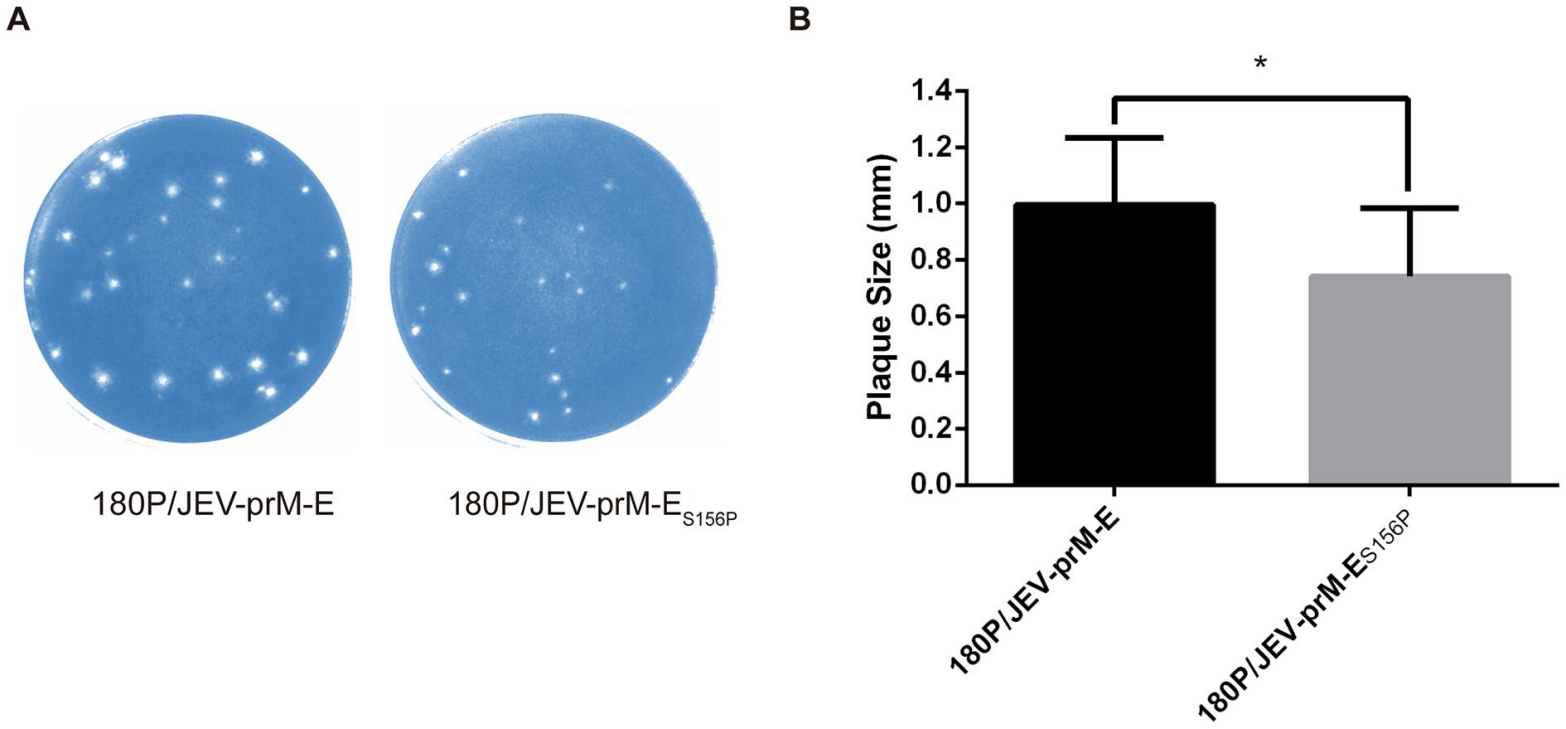
The plaque morphology of the 180P/JEV-prM-E_S156P_ and its parental virus. **(A)** 180P/JEV-prM-E_S156P_ and its parental virus 180P/JEV-prM-E pattern morphological observation. **(B)** 180P/JEV-prM-E_S156P_ and its parental virus plaque diameter. ^*^p < 0.05.

Plaques assay showed that plaque of 180P/JEV-prM-E_S156P_ was smaller than that of the 180P/JEV-prM-E (Figures 4A,B), which suggested that the absence of the glycosylation site reduced the plaque formation ability of the virus, indicating further attenuation of the virulence of 180P/JEV-prM-E_S156P_ virus.

To further investigate the impact of the E S156P mutation on the pathogenicity of the virus, a mouse experiment was conducted. The results showed that mice infected intraperitoneally (i.p.) or intranasally (i.n.) with either 180P/JEV-prM-E_S156P_ or JEV vaccine strain SA14-14-2 did not experience any weight loss or death (Figures 2A,B). In contrast, the 180P/JEV-prM-E caused weight loss and 20% mortality in the i.n. group, which indicated that the mutation of the glycosylation site of E protein further weakened the neuroinvasiveness of the 180P/JEV-prM-E_S156P_. In the i.c. infected group, both 180P/JEV-prM-E_S156P_ and JEV vaccine strain SA14-14-2 only caused slight weight loss, and none of the mice died (Figure 2C). In contrast, the 180P/JEV-prM-E caused severe weight loss and 60% mortality (Figure 2C). All the data indicated that the mutation of glycosylation site of the E protein further reduced the neurovirulence of the 180P/JEV-prM-E_S156P_ virus.

The virus distribution data indicated neither the 180P/JEV-prM-E_S156P_ nor JEV vaccine strain SA14-14-2 were detected in any tissues of intraperitoneally (i.p.) or intranasally (i.n.) infected mice (Figures 3A,B). However, a lower titer of 180P/JEV-prM-E was detected in the brain of one i.n. infected mouse on 8 dpi (Figure 3B). In the i.c. infected group, the viral titers of the 180P/JEV-prM-E_S156P_ group were significantly lower than those of the 180P/JEV-prM-E virus in brains on both 4 and 8 dpi (Figure 3C), which suggests that the virulence of the 180P/JEV-prM-E_S156P_ is significantly reduced. These results suggested that the glycosylation site of the E protein is critical for JEV virulence.

### 3.4. 180P/JEV-prM-E_S156P_ provided protection against challenge with wildtype JEV in mouse model

**Figure 5:**
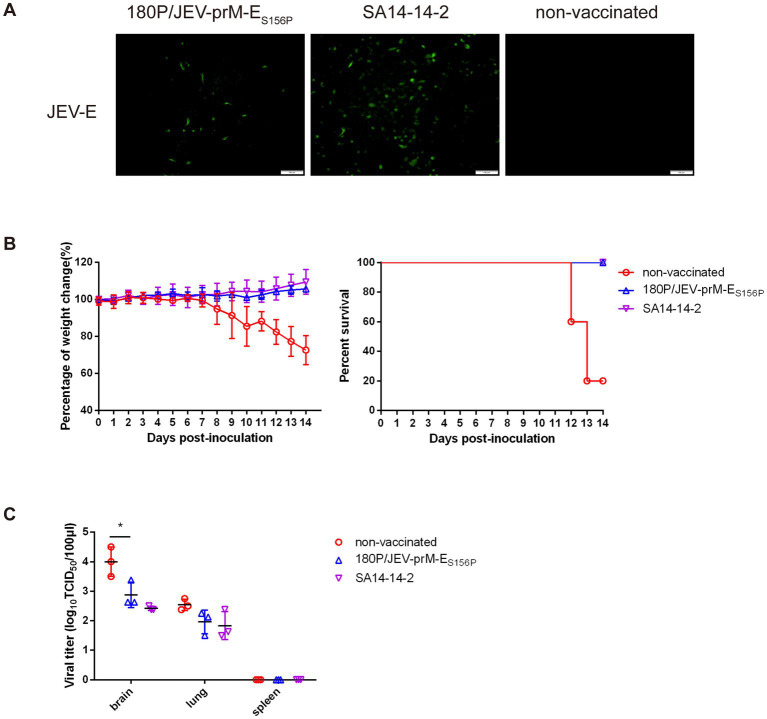
180P/JEV-prM-E_S156P_ provided protection against challenge with wildtype JEV in mouse model. **(A)** The detection of JEV E protein antibody after immunization with 180P/JEV-prM-E_S156P_ chimeric virus and SA14-14-2 vaccine by IFA. **(B)** Percentage of weight change and percent survival of three group after challenge with wildtype JEV. **(C)** Viral titers in brain, spleen and lung of challenge mice were determined on BHK-21 cells at 6 dpi. Body weight and survival data of mice in each group were recorded daily until to 14 dpi. ^*^p < 0.05.

To explore the efficacy of 180P/JEV-prM-E_S156P_ virus, the vaccination-challenge experiment was conducted in mouse model. On the 21st day after immunization, the serum of mice was analyzed, and the results showed that antibodies against JEV E protein were detected in both the 180P/JEV-prM-E_S156P_ chimeric virus group and the SA14-14-2 vaccine immunized group (Figure 5A) while the antibodies against TMUV C, prM, and E protein was not detected in the 180P/JEV-prM-E_S156P_ chimeric virus group. This result indicates that chimeric viruses induced antibodies specific against JEV E protein in mice.

The challenge results showed that there was no weight loss or death in the 180P/JEV-prM-E_S156P_ and JEV vaccine strain SA14-14-2 vaccinated mice after challenge with the wildtype JEV (Figure 5B). Whilst, the wildtype JEV caused obvious clinical symptoms such as fluffy hair, depression, loss of appetite and weight lost around 7 days after challenge and 80% mortality in the non-vaccinated group within 14 dpi (Figure 5B). The virus distribution data showed that 180P/JEV-prM-E_S156P_ and JEV vaccine SA14-14-2 immunized mice had lower virus load than the non-vaccinated group after challenge with the wildtype JEV in brain (Figure 5C). These results showed that although the 180P/JEV-prM-E_S156P_ and JEV vaccine SA14-14-2 immunization could not completely prevent wildtype JEV from invading the brain tissue of mice, but the replication ability of the wildtype JEV in the brain was significantly reduced, and the mice were completely protected from death caused by the wildtype JEV challenge.

## 4. Discussion

Since the outbreak of TMUV in China in 2010, it has caused significant economic losses to the duck industry. To prevent the disease, a live attenuated DTMUV vaccine FX2010-180P was licensed in China in 2018. The experiment results showed that the vaccine strain was attenuated in both of avian and mammalian models. The FX2010-180P induced strong immune response and completely protected the immunized animals from infection. All the result suggested that the FX2010-180P could be a backbone for developing vaccines of other Flaviviruses by switching its main structural proteins.

Flaviviruses pose a serious threat to humans and animals, and there is currently no specific treatment for the diseases caused by flaviviruses. Vaccination is the primary method to prevent these diseases. Currently, vaccines against flaviviruses are broadly divided into three types: inactivated vaccines, live attenuated vaccines, and live yellow fever virus (YFV) recombinant vaccines against JEV (Chambers et al., 1999; Guirakhoo et al., 1999) and DENV (Guirakhoo et al., 2000). These recombinant flavivirus vaccines were constructed by replacing the prM and E genes of the YF vaccine strain with those of other flavivirus, such as DENV and WNV (Chambers et al., 1999; Guirakhoo et al., 1999, 2000; Bonaldo et al., 2000; Monath et al., 2000; Caufour et al., 2001; Chambers et al., 2003; Arroyo et al., 2004; Galler et al., 2005).

Japanese encephalitis virus is still a threat to the public health, according to data from the Chinese Bureau of Disease Control and Prevention, between 2009 and 2021, China had reported 18,597 cases of JEV infection, resulting in 757 deaths and a mortality rate of 4.1%. The flavivirus E protein is a main structural protein that plays an important role in binding and entry, and prM/E proteins are the main neutralizing antigens of JEV (Konishi et al., 1998). Studies of JEV attenuated vaccine (SA14-14-2) showed that substitutions of amino acids L107F, E138K, I176V, T177A, E244G, Q264H, K279M, A315V, S366A, and K439R in the E protein can attenuate the neurovirulence of JEV (Yu, 2010; Gromowski et al., 2015). Moreover, the glycosylation of the E protein, as a glycoprotein, also has significant impact on viral replication and virulence (Liang et al., 2018). JEV E protein has only one N-linked glycosylation site on N154, which is crucial for the virulence of JEV. Deletion of the N154 glycosylation site (N154A) significantly attenuated the viral replication in cultured cells, and reduces neurovirulence and neuroinvasiveness in challenged mice. These results indicate that the glycosylation has a significant effect on the virulence of JEV.

In this study, we investigated whether 180P could be used as a backbone for developing flavivirus vaccines, the chimeric virus 180P/JEV-prM-E and 180P/JEV-prM-E_S156P_ with the potential glycosylation site deletion were rescued successfully. In the mouse model, the neurovirulence and neuroinvasiveness of the chimeric virus 180P/JEV-prM-E was significantly decreased compared with the wildtype JEV. However, the 180P/JEV-prM-E is still more virulent than the parental 180P. Notably, when the glycosylation site was deleted in the 180P/JEV-prM-E_S156P_ E protein, the neurovirulence was further reduced and neuroinvasiveness was completely abolished. These finding suggest that the absence of the glycosylation site further weakens the 180P/JEV-prM-E_S156P_ virus. And 180P/JEV-prM-E_S156P_ could provide complete protection against challenge with wildtype JEV in mouse model. However, the limitation of this study is that although point mutation virus 180P/JEV-prM-E_S156P_ could protect immunized mice from death after challenge, it cannot prevent wildtype JEV replication in the brain. Overall, in this study, we demonstrated that 180P can be used as a backbone to develop JEV attenuated vaccine candidate, which provided a new vector tool for the development and preparation of flavivirus vaccines.

## Data availability statement

The raw data supporting the conclusions of this article will be made available by the authors, without undue reservation.

## Ethics statement

The animal study was reviewed and approved by Institutional Animal Care and Use Committee of Shanghai Veterinary Research Institute (SHVRI) with permit number was SHVRI-SZ-20200506-02.

## Author contributions

BX and XL conceived of the study and participated in its design and coordination. BX participated in laboratory work with the help of DY, QT, CY, and ZZ. BX drafted the manuscript and ZL and QL modified it. ZL directed the project. All authors contributed to the article and approved the submitted version.

## Funding

This work was supported by the National Natural Science Foundation of China (32102654 and 32072862), the Shanghai Agriculture Applied Technology Development Program, China (2021-02-08-00-12-F00746), the Shanghai Sailing Program (no. 21YF1456500), and the Agricultural Science and Technology Innovation Program (CAAS-ZDRW202203).

## Conflict of interest

The authors declare that the research was conducted in the absence of any commercial or financial relationships that could be construed as a potential conflict of interest.

## Publisher’s note

All claims expressed in this article are solely those of the authors and do not necessarily represent those of their affiliated organizations, or those of the publisher, the editors and the reviewers. Any product that may be evaluated in this article, or claim that may be made by its manufacturer, is not guaranteed or endorsed by the publisher.

## References

[ref1] Allison S. L. Schalich J. Stiasny K. Mandl C. W. Heinz F. X. (2001). Mutational evidence for an internal fusion peptide in flavivirus envelope protein E. J. Virol. 75, 4268–4275. doi: 10.1128/jvi.75.9.4268-4275.2001, PMID: 11287576 PMC114172

[ref2] Arroyo J. Miller C. Catalan J. Myers G. A. Ratterree M. S. Trent D. W.. (2004). ChimeriVax-West Nile virus live-attenuated vaccine: preclinical evaluation of safety, immunogenicity, and efficacy. J. Virol. 78, 12497–12507. doi: 10.1128/JVI.78.22.12497-12507.2004, PMID: 15507637 PMC525070

[ref3] Bonaldo M. C. Caufour P. S. Freire M. S. Galler R. (2000). The yellow fever 17D vaccine virus as a vector for the expression of foreign proteins: development of new live flavivirus vaccines. Mem. Inst. Oswaldo Cruz 95, 215–223. doi: 10.1590/s0074-02762000000700037, PMID: 11142718

[ref4] Cao Z. Zhang C. Liu Y. Liu Y. Ye W. Han J.. (2011). Tembusu virus in ducks, China. Emerg. Infect. Dis. 17, 1873–1875. doi: 10.3201/eid1710.101890, PMID: 22000358 PMC3310660

[ref5] Carbaugh D. L. Lazear H. M. (2020). Flavivirus envelope protein glycosylation: impacts on viral infection and pathogenesis. J. Virol. 94:e00104. doi: 10.1128/JVI.00104-20, PMID: 32161171 PMC7269438

[ref6] Caufour P. S. Motta M. C. A. Yamamura A. M. Y. Vazquez S. Ferreira I. I. Jabor A. V.. (2001). Construction, characterization and immunogenicity of recombinant yellow fever 17D-dengue type 2 viruses. Virus Res. 79, 1–14. doi: 10.1016/s0168-1702(01)00273-8, PMID: 11551641

[ref7] Chambers T. J. Hahn C. S. Galler R. Rice C. M. (1990). Flavivirus genome organization, expression, and replication. Annu. Rev. Microbiol. 44, 649–688. doi: 10.1146/annurev.mi.44.100190.003245, PMID: 2174669

[ref8] Chambers T. J. Liang Y. Droll D. A. Schlesinger J. J. Davidson A. D. Wright P. J.. (2003). Yellow fever virus/dengue-2 virus and yellow fever virus/dengue-4 virus chimeras: biological characterization, immunogenicity, and protection against dengue encephalitis in the mouse model. J. Virol. 77, 3655–3668. doi: 10.1128/jvi.77.6.3655-3668.2003, PMID: 12610141 PMC149507

[ref9] Chambers T. J. Nestorowicz A. Mason P. W. Rice C. M. (1999). Yellow fever/Japanese encephalitis chimeric viruses: construction and biological properties. J. Virol. 73, 3095–3101. doi: 10.1128/JVI.73.4.3095-3101.1999, PMID: 10074160 PMC104070

[ref10] Galler R. Marchevsky R. S. Caride E. Almeida L. F. C. Yamamura A. M. Y. Jabor A. V.. (2005). Attenuation and immunogenicity of recombinant yellow fever 17D-dengue type 2 virus for rhesus monkeys. Braz. J. Med. Biol. Res. 38, 1835–1846. doi: 10.1590/s0100-879x2005001200012 16302098

[ref11] Gromowski G. D. Firestone C. Y. Whitehead S. S. (2015). Genetic determinants of Japanese encephalitis virus vaccine strain SA14-14-2 that govern attenuation of virulence in mice. J. Virol. 89, 6328–6337. doi: 10.1128/JVI.00219-15, PMID: 25855730 PMC4474313

[ref12] Guirakhoo F. Weltzin R. Chambers T. J. Zhang Z. X. Soike K. Ratterree M.. (2000). Recombinant chimeric yellow fever-dengue type 2 virus is immunogenic and protective in nonhuman primates. J. Virol. 74, 5477–5485. doi: 10.1128/jvi.74.12.5477-5485.2000, PMID: 10823852 PMC112032

[ref13] Guirakhoo F. Zhang Z.-X. Chambers T. J. Delagrave S. Arroyo J. Barrett A. D. T.. (1999). Immunogenicity, genetic stability, and protective efficacy of a recombinant, chimeric yellow fever-Japanese encephalitis virus (ChimeriVax-JE) as a live, attenuated vaccine candidate against Japanese encephalitis. Virology 257, 363–372. doi: 10.1006/viro.1999.9695, PMID: 10329547

[ref14] Konishi E. Yamaoka M. Khin-Sane-Win K. I. Mason P. W. (1998). Induction of protective immunity against Japanese encephalitis in mice by immunization with a plasmid encoding Japanese encephalitis virus premembrane and envelope genes. J. Virol. 72, 4925–4930. doi: 10.1128/JVI.72.6.4925-4930.1998, PMID: 9573260 PMC110053

[ref15] Leng S. L. Huang R. Feng Y. N. Peng L. J. Yang J. Li Y. H. (2020). The pre membrane and envelope protein is the crucial virulence determinant of Japanese encephalitis virus. Microb. Pathog. 148:104492. doi: 10.1016/j.micpath.2020.104492, PMID: 32916243

[ref16] Leung J. Y. Pijlman G. P. Kondratieva N. Hyde J. Mackenzie J. M. Khromykh A. A. (2008). Role of nonstructural protein NS2A in flavivirus assembly. J. Virol. 82, 4731–4741. doi: 10.1128/JVI.00002-08, PMID: 18337583 PMC2346727

[ref17] Li G. Gao X. Xiao Y. Liu S. Peng S. Li X.. (2014). Development of a live attenuated vaccine candidate against duck Tembusu viral disease. Virology 450–451, 233–242. doi: 10.1016/j.virol.2013.12.028, PMID: 24503086

[ref18] Liang J. J. Chou M. W. Lin Y. L. (2018). DC-SIGN binding contributed by an extra N-linked glycosylation on Japanese encephalitis virus envelope protein reduces the ability of viral brain invasion. Front. Cell. Infect. Microbiol. 8:239. doi: 10.3389/fcimb.2018.00239, PMID: 30042931 PMC6048278

[ref19] Lorenz I. C. Allison S. L. Heinz F. X. Helenius A. (2002). Folding and dimerization of tick-borne encephalitis virus envelope proteins prM and E in the endoplasmic reticulum. J. Virol. 76, 5480–5491. doi: 10.1128/jvi.76.11.5480-5491.2002, PMID: 11991976 PMC137023

[ref20] Mackenzie J. S. Gubler D. J. Petersen L. R. (2004). Emerging flaviviruses: the spread and resurgence of Japanese encephalitis, West Nile and dengue viruses. Nat. Med. 10, S98–S109. doi: 10.1038/nm1144, PMID: 15577938

[ref21] Miller S. Kastner S. Krijnse-Locker J. Buhler S. Bartenschlager R. (2007). The non-structural protein 4A of dengue virus is an integral membrane protein inducing membrane alterations in a 2K-regulated manner. J. Biol. Chem. 282, 8873–8882. doi: 10.1074/jbc.M609919200, PMID: 17276984

[ref22] Monath T. P. Levenbook I. Soike K. Zhang Z. X. Ratterree M. Draper K.. (2000). Chimeric yellow fever virus 17D-Japanese encephalitis virus vaccine: dose-response effectiveness and extended safety testing in rhesus monkeys. J. Virol. 74, 1742–1751. doi: 10.1128/jvi.74.4.1742-1751.2000, PMID: 10644345 PMC111650

[ref23] Monath T. P. Vasconcelos P. F. (2015). Yellow fever. J. Clin. Virol. 64, 160–173. doi: 10.1016/j.jcv.2014.08.030 25453327

[ref24] Pierson T. C. Diamond M. S. (2020). The continued threat of emerging flaviviruses. Nat. Microbiol. 5, 796–812. doi: 10.1038/s41564-020-0714-0 32367055 PMC7696730

[ref25] Platt G. S. Way H. J. Bowen E. T. Simpson D. I. Hill M. N. Kamath S.. (1975). Arbovirus infections in Sarawak, October 1968--February 1970 Tembusu and Sindbis virus isolations from mosquitoes. Ann. Trop. Med. Parasitol. 69, 65–71. doi: 10.1080/00034983.1975.11686984 235907

[ref26] Su J. Li S. Hu X. Yu X. Wang Y. Liu P.. (2011). Duck egg-drop syndrome caused by BYD virus, a new Tembusu-related flavivirus. PLoS One 6:e18106. doi: 10.1371/journal.pone.0018106, PMID: 21455312 PMC3063797

[ref27] Tang Y. Diao Y. Yu C. Gao X. Ju X. Xue C.. (2013). Characterization of a Tembusu virus isolated from naturally infected house sparrows (*Passer domesticus*) in northern China. Transbound. Emerg. Dis. 60, 152–158. doi: 10.1111/j.1865-1682.2012.01328.x, PMID: 22515847

[ref28] Thontiravong A. Ninvilai P. Tunterak W. Nonthabenjawan N. Chaiyavong S. Angkabkingkaew K.. (2015). Tembusu-related Flavivirus in ducks, Thailand. Emerg. Infect. Dis. 21, 2164–2167. doi: 10.3201/eid2112.150600, PMID: 26584133 PMC4672441

[ref29] Umareddy I. Chao A. Sampath A. Gu F. Vasudevan S. G. (2006). Dengue virus NS4B interacts with NS3 and dissociates it from single-stranded RNA. J. Gen. Virol. 87, 2605–2614. doi: 10.1099/vir.0.81844-0, PMID: 16894199

[ref30] Yan D. Shi Y. Wang H. Li G. Li X. Wang B.. (2018). A single mutation at position 156 in the envelope protein of Tembusu virus is responsible for virus tissue tropism and transmissibility in ducks. J. Virol. 92:e00427. doi: 10.1128/JVI.00427-18, PMID: 29899104 PMC6096821

[ref31] Yan D. Wang B. Shi Y. Ni X. Wu X. Li X.. (2022). A single mutation at position 120 in the envelope protein attenuates Tembusu virus in ducks. Viruses 14:447. doi: 10.3390/v14030447, PMID: 35336854 PMC8951291

[ref32] Yan P. Zhao Y. Zhang X. Xu D. Dai X. Teng Q.. (2011). An infectious disease of ducks caused by a newly emerged Tembusu virus strain in mainland China. Virology 417, 1–8. doi: 10.1016/j.virol.2011.06.003 21722935

[ref33] Yu Y. X. (2010). Phenotypic and genotypic characteristics of Japanese encephalitis attenuated live vaccine virus SA14-14-2 and their stabilities. Vaccine 28, 3635–3641. doi: 10.1016/j.vaccine.2010.02.105, PMID: 20226891

[ref34] Zhang L. Zhao D. Han K. Huang X. Liu Y. Liu Q.. (2020). Tembusu virus enters BHK-21 cells through a cholesterol-dependent and clathrin-mediated endocytosis pathway. Microb. Pathog. 147:104242. doi: 10.1016/j.micpath.2020.104242, PMID: 32407862

